# Comparison of W. Arnett’s cephalometric analysis measurements performed using artificial intelligence and manual measurements

**DOI:** 10.1007/s00784-026-06915-7

**Published:** 2026-05-11

**Authors:** Betul Kula, Elif Nadide Akay Polat

**Affiliations:** 1 Department of Orthodontics, Faculty of Dentistry, Istanbul Galata University, Evliya Celebi Street, Mesrutiyet Boulevard No: 62, Beyoglu, Istanbul, Turkey; 2 Department of Orthodontics, Faculty of Dentistry, Istanbul Medeniyet University, Istanbul, Turkey

**Keywords:** Artificial intelligence, WebCeph™, Lateral Cephalometry, Arnett Analysis

## Abstract

**Objectives:**

This study aimed to compare the linear and angular Arnett cephalometric measurements obtained from WebCeph Premium™ with those from manual cephalometric analysis and to assess the reliability of the automated measurements.

**Methods:**

Thirty-two pre-treatment lateral cephalograms of patients were randomly selected. Images were calibrated, 29 landmarks were manually traced, and 40 parameters were recorded by two orthodontists (6 angular, 34 linear). WebCeph Premium™ (version 2.0.0, AssembleCircle Corp., Gyeonggi-do, Republic of Korea) automatically identified landmarks and performed Arnett’s cephalometric analysis. After 15 days, orthodontists reassessed the radiographs. Reliability and repeatability were evaluated using the intraclass correlation coefficient (ICC), which exceeded 0.95, indicating excellent agreement. Normality was assessed using the Shapiro–Wilk test, and group comparisons were performed with paired t-tests.

**Results:**

For Researcher 1, initial ICC values ranged from 0.003 to 0.984 and final values from 0.617 to 0.996. For Researcher 2, initial ICC values ranged from 0.022 to 0.999 and final values from 0.601 to 0.999. Inter-observer ICC values ranged from 0.03 to 0.984, with most measurements showing high agreement. Manual tracing and WebCeph Premium™ showed high agreement, with ICC values ranging 0.913 and 0.995.

**Conclusions:**

WebCeph Premium™ demonstrates high concordance with manual Arnett analyses; however, further refinement of certain parameters is necessary to enhance AI precision.

**Clinical Relevance:**

The utilization of WebCeph Premium™ within clinical settings has the capacity to enhance the efficiency of cephalometric analysis for orthognathic cases by minimizing manual workload and inter-examiner variability. This can result in more consistent and efficient treatment planning.

**Supplementary Information:**

The online version contains supplementary material available at 10.1007/s00784-026-06915-7.

## Introduction

Cephalometric analysis is a crucial tool in the field of orthodontics, providing invaluable insights for diagnosis, treatment planning, assessing growth changes, and evaluating of treatment outcomes [1]. It facilitates the identification of key cephalometric landmarks, including skeletal, dental, and soft tissue points, as well as the measurement of distances, angles, and planes between them [2]. To analyze these radiographs effectively, key anatomical structures must be identified through landmark identification and manual tracing.

While regarded as the gold standard, manual cephalometric analysis presents significant challenges, time-intensive procedures that require specialist expertise, and vulnerability to human error due to fatigue and measurement inaccuracies [3]. These limitations have driven the development of computerized analysis systems as technological alternatives [4].

Cephalometric analysis has undergone a shift in its basic assumptions from manual to digital methods, thereby revolutionizing orthodontic practice [5]. Digital cephalometric tracing offers substantial advantages over manual techniques by providing a time-efficient and highly accurate method for analyzing cephalometric radiographs. This approach effectively mitigates human error, relying on sophisticated computer algorithms to identify landmarks and perform calculations with unparalleled precision. In addition to being time-efficient, thereby reducing the workload of clinicians, this technology integrates advanced artificial intelligence (AI) capabilities, facilitating the automatic identification of landmarks and the immediate computation of measurements, which streamlines the entire diagnostic process [4–7]. Digital archiving ensures long-term preservation of images without the risk of deterioration and facilitates easy sharing of digital files. Digital cephalometric tracing is a powerful tool for orthodontic diagnostics [8].

In recent years, the application of AI and digital technologies in healthcare has enabled such analyses to be performed with greater efficiency, reliability and reproducibility. AI-based software, for instance WebCeph Premium™ (AssembleCircle Corp., Gyeonggi-do, Republic of Korea), has become an alternative to manual methods in orthodontic planning, including the assessment of factors related to facial aesthetics. These software programs have been designed to minimize analysis time while maintaining the lowest possible error rates. They enable rapid execution of multiple cephalometric analyses once landmarks are accurately delineated on digital cephalogram images [9].

Landmark determination is a primary source of inaccuracy in cephalometric analysis, thereby driving the development of AI-based software, such as WebCeph Premium™ which is a web-based, fully automated platform that offers nine cephalometric and two composite analyses, along with the interpretation of results. The software’s functionality encompasses automated tracing, superimposition, treatment simulation, photo archiving, and manual landmark adjustment with automatic recalculation [10].

WebCeph Premium™ is a software program that can perform cephalometric analyses, including the Arnett analysis. Although numerous cephalometric analysis methods are currently in use, Arnett analysis is a widely accepted approach globally, particularly as a standard in orthognathic surgery diagnosis and treatment planning [11]. Furthermore, Arnett cephalometric analysis is prominent for its aesthetic evaluation and functional balance, utilizing soft tissue-oriented approaches [12]. To the best of our knowledge, despite the clinical and scientific importance of Arnett cephalometric analysis, only one study has employed AI-supported software in this context. A review of the literature revealed a lack of studies evaluating the accuracy of Arnett cephalometric analysis measurements using WebCeph Premium™ software.

Despite the continuous advancements in this domain, automatic cephalometric landmark identification still struggles to achieve consistent accuracy across all landmarks, underscoring the need for further research and development in this field.

Another aim of this study is to determine whether Arnett analysis, a tool used in orthodontic treatment and orthognathic surgery planning that has the potential to induce significant facial alterations, contributes to the clinical success of practitioners by enabling faster and more streamlined procedures through the use of AI assistance.

The null hypothesis suggests that there is no significant difference between the linear and angular Arnett cephalometric measurements obtained from WebCeph Premium™ and those derived from manual analysis.

## Materials and methods

This study was registered and approved by the Human Research Ethics Committee of Istanbul Medeniyet University with the decision number 2023/0426. The sample size was calculated using the methodology of previous studies by Alqahtani and Silva et al. [13, 14].

A sample size of 32 was calculated (Intraclass correlation coefficient ICC = 0.70, 95% power, 5% α), with 32 cephalograms were randomly selected to compensate for potential data loss. No distinction was made in terms of gender, type of malocclusion and/or skeletal pattern. A balanced cohort of 32 patients was included to ensure methodological rigor, but the analyses focused on the entire group to assess the overall accuracy of AI predictions.

Inclusion criteria.

X-rays of sufficient quality to enable the identification of the necessary anatomical landmarks.

Absence of unerupted or partially erupted teeth obstructing the required anatomical points.

Patients over 16 years of age.

Exclusion criteria.

Cephalograms with poor image quality.

Cephalograms with positional errors caused by ear rod markers.

Cephalograms with motion artefacts, uneven resolution or poor contrast.

Individuals with severe craniofacial deformities or facial asymmetries.

Lateral cephalograms of 32 individuals were obtained under standardized conditions using a single imaging device. The Planmeca Proline PM-2002 model has an anode-subject distance of five steps. The tube voltage was 70 kVp, the current was 12 mA, and the exposure time was 1.8 s. Cephalograms were obtained with a calibration chart to determine magnification. All images were calibrated with two points on the ruler. The 32 lateral cephalograms used in the study were captured at a maximum size of 2272 × 2045 pixels, with a resolution of 217 dpi, in grayscale, and at 0% magnification. These files were saved in the JPEG format. The cephalograms were subsequently stored and analyzed on a personal computer (GRUNDIG RMU5A2D Intel(R) Core (TM) i5-5200U CPU@ 2.20 GHz 2.20 GHz).

### Cephalometric analysis

#### Manual tracing

**Fig. 1:**
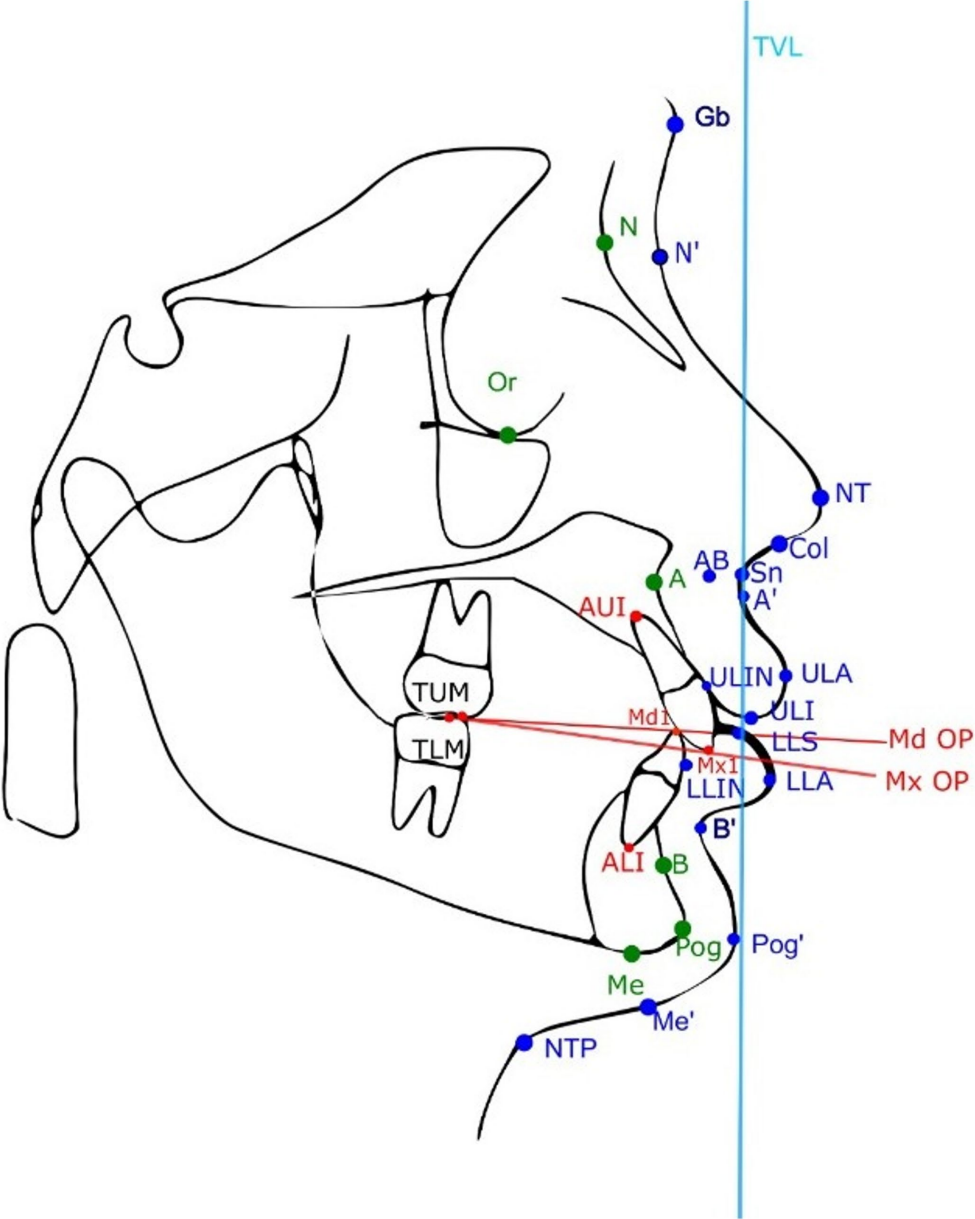
Reference points and planes used in arnett soft tissue cephalometric analysis

All radiographs were evaluated in a dimly lit room under standardized lighting conditions and calibrated manually using the same calibrated monitor to minimize visual bias in landmark identification. The patients’ pre-treatment lateral cephalograms were manually traced, a total of 29 landmarks were identified, including 17 soft tissue points, 6 skeletal points, and 6 dental points. (Fig. 1). Subsequently, cephalometric measurements of 40 parameters (6 angular and 34 linear) were recorded (Table 1). Manual cephalometric measurements were performed independently by two experienced orthodontists (B.K.-10 years, E.P.-9 years) (Supplementary file). During the analysis period, the clinicians were blinded to both the AI-generated results and the measurements of each other. A maximum of 5 cephalometric analysis was performed per day to minimize errors due to fatigue. In order to avoid the risk of memorizing landmarks (recall bias), two orthodontists repeated all measurements following a minimum interval of 15 days after the initial evaluation. Linear and angular measurements were performed after tracing the required planes and angles.

**Table 1:** Skeletal, dental, soft tissue parameters and planes used in Arnett analysis

SKELETAL PARAMETERS	Abbreviations	Explanations
Nasion	N	It is the most anterior point of the frontonasal suture where the frontal and nasal bones meet.
Orbitale	Or	The most inferior point on the infraorbital margin.
Point A	A	The deepest point in the concavity of the anterior maxilla between the anterior nasal spine and the alveolar crest.
Point B	B	The deepest point in the concavity of the anterior mandible between the alveolar crest and pogonion.
Pogonion	Pog	The most anterior point on the bony chin
Menton	Me	The most inferior point on the bony chin
DENTAL PARAMETERS		
Maxillary central incisor tip	Mx 1	The most inferior point on the tip of the upper incisor.
Apex of upper incisor	AUI	The most superior point on the tip of the upper incisor apex.
Mandibular central incisor tip	Md 1	The most superior point on the tip of the lower incisor.
Apex of lower incisor	ALI	The most inferior point on the tip of the upper incisor apex.
Tip of upper molar	TUM	The most inferior point on the mesio buccal cusp of the upper molar
Tip of lower molar	TLM	The most superior point on the mesio buccal cusp of the lower molar
SOFT TISSUE PARAMETERS		
Glabella	Gb	Most anterior point on the soft tissue contour of the forehead
Soft tissue nasion	Na’	Most posterior point on the soft tissue contour on the area of the frontonasal suture
Columella	Col	Point on the lower surface of the nose, determining the anterior limit of the nasolabial angle
Orbital rim	OR’	The intersection of the skeletal orbital rim with the vertical line passing through the pupil when the patient is looking straight ahead
Subpupil	SP	Simulated directly below the straight-ahead gaze of the pupil. Vertically, the subpupil marker was placed one half the vertical distance between the orbital rim and alar base markers.
Cheekbone	CB	The intersection of the right malar height of contour and a vertical line through outer canthus.
Alar base	AB	The deepest depression at the alar base of the nose
Nasal tip	NT	The most anterior point of nasal tip
Subnasale	Sn	Midpoint on the nasolabial soft tissue contour between the columellae crest and upper lip.
Soft tissue point A	A’	The deepest point in the concavity between the subnasale and the upper lip anterior.
Upper Lip Anterior	ULA	The most anterior point on the convexity of upper lip
Upper lip inferior	ULI	The most inferior point on the upper lip
Lower lip superior	LLS	The most superior point on the lower lip
Lower lip anterior	LLA	The most anterior point on the convexity of lower lip
İnner part of the upper lip	ULIN	The inner point of the upper lip in contact with the front surface of the upper incisor.
İnner part of the lower lip	LLİN	The inner point of the lower lip in contact with the front surface of the lower incisor.
Soft Tissue Point B	B’	The deepest point in the concavity between the lower lip anterior and the soft tissue pogonion.
Soft Tissue pogonion Pog’	Pog’	The most anterior point on the upper lip
Neck-Throat Point	NTP	Intersection of the subnasale-soft tissue pogonion and the throat line.
Soft Tissue Menton	Me’	The most inferior point on the soft tissue chin.
PLANES		
True Vertical Line	TVL	The vertical line passing through the subnasale when the patient’s head is in the natural head position.
Mx Occlusal Plane	Mx OP	The mesiobuccal cusp tip of the maxillary permanent first molar to maxillary incisor tip.
Md Occlusal Plane	Md OP	The mesiobuccal cusp tip of the mandibular permanent first molar to mandibular incisor tip.
ANGULAR MEASUREMENTS		
Mx occlusal plane to TVL		Angle between maxillary occlusal planet to true vertical line
Mx1 to Mx occlusal Plane		Angle between maxillary incisor tip and maxillary occlusal plane
Md1 to Md occlusal Plane		Angle between mandibular incisor tip and mandibular occlusal plane
Nasolabial angle	NLA	Angle between columella, subnasale, upper lip anterior.
Upper Lip Angle		Angle between upper lip angle, subnasale and true vertical line.
Facial angle		Angle between soft tissue glabella, subnasale and soft tissue pogonion
LINEAR MEASUREMENTS		
Overjet		Horizontal distance between the incisal edge of the upper and lower incisors
Overbite		Vertical overlap of the maxillary incisors over the mandibular incisors when the jaws are in occlusion
Upper lip thickness		Short distance between the labial surface of the upper central incisor and the upper lip anterior.
Lower lip thickness		The shortest distance between the labial surface of the lower central incisor and Lower lip anterior.
Pogonion-pogonion’		Horizontal distance between pogonion and soft tissue pogonion.
Menton- Menton’		Horizontal distance between menton and soft tissue menton.
Nasion- menton		Horizontal distance between nasion and menton.
Upper lip length		Perpendicular distance from the Subnasale to-Upper lip inferior.
İnterlabial gap		Perpendicular distance between upper lip inferior and lower lip superior.
Lower lip length		Perpendicular distance between lower lip superior to soft tissue menton.
Lower 1/3 of face		Perpendicular distance from the subnasale to soft tissue menton.
Mx1 exposure		Upper incisor exposure is the vertical distance between the points upper lip inferior and maxillary central incisor tip.
Maxillary height		Perpendicular distance between subnasale and maxillary central incisor tip.
Mandibular height		Perpendicular distance between mandibular central incisor tip and soft tissue menton.
Projection to TVL (glabella, orbital rims, cheekbone, subpupil, alar base, nasal projection, subnasale, A point’, upper lip anterior’, Mx1, Mn 1, lower lip anterior’, B point’, pogonion’),		Horizontal distance of the specified points to the TVL plane.
G’ to A’, G’ to Pog’, OR’ to Pog’, Sn to Pog’, A’ to B’, ULA to LLA, Md1 to Pog’, LLA to Pog’, B’to Pog’, NTP to Pog’		Horizontal distance between indicated points.

The orbital rim (OR’), cheekbones (CB), subpupil (SP), orbital rim-soft tissue A point (Or’-A’), orbital rim-soft tissue pogonion (Or’-Pog’) measurements were excluded from the study because no metallic markers had been placed on the previously obtained X-rays. Although the WebCeph Premium™ software automatically identifies 32 landmarks as part of the W. Arnett analysis template, only 29 of these points were manually traced and included in the statistical comparison to ensure methodological consistency. Consequently, a comparison with the AI application was not possible.

The calibration of each image to its actual size in millimeters was performed by measuring the known distance (10 mm) between two fixed points on the ruler visible in the cephalogram. When applicable, the magnification of the images was calculated, and the final values were obtained by applying the magnification factor to the linear measurements. All measurements were recorded into a Microsoft Office Excel 2007 spreadsheet (Microsoft Corp., Redmond, Washington, USA), and the resulting numerical values were considered as the control.

#### Digital tracing

**Fig. 2:**
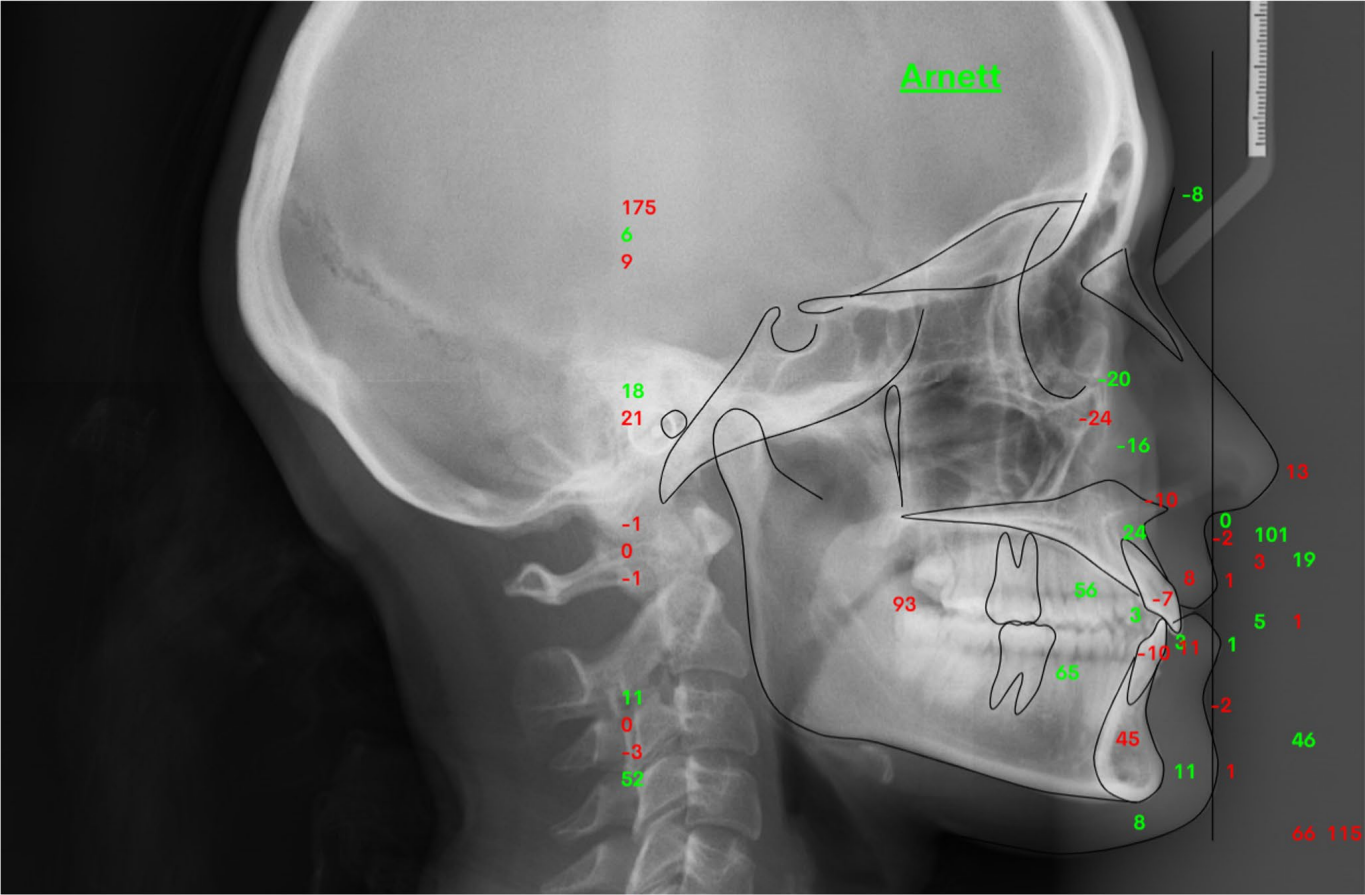
Cephalometric marking according to Arnett analysis performed by WebCeph™

ital tracingCephalometric analysis of the X-rays was performed using WebCeph Premium™ (https://webceph.com, AssembleCircle Corp., Gyeonggi-do, Republic of Korea). The software required the creation of an online account, through which patient profiles were established and digital cephalogram images uploaded to the corresponding profiles. Arnett’s analysis was then selected from the available cephalometric analysis options. The software subsequently performed calibration using AI, automatic landmark identification and tracking using the digitization feature (Fig. 2).

The image was calibrated using the 10 mm ruler displayed on the screen, which was aligned with the calibration ruler embedded in the digital cephalogram. Finally, the cephalometric measurement values were downloaded as PDFs and entered into the Microsoft Office Excel™ spreadsheet used for manual tracking values (Supplementary files 1–2). This procedure was performed on all 32 digital cephalograms.

In order to evaluate the reliability of measurement in both the control and test groups, radiographs were deleted from the system memory 15 days following the initial recordings. This allowed re-evaluation by both the examiner and WebCeph Premium™, thereby enhancing reproducibility and reliability. Subsequently, the measurements were repeated to further ensure consistency, and the resulting numerical values were recorded in an Excel™ spreadsheet for statistical analysis.

### Statistical analysis

The data were analyzed using IBM SPSS V23. The Shapiro-Wilk test was used to analyze compliance with normal distribution. Inter-observer and intra-observer agreement were analyzed using the ICC. Intra-observer agreement, which assesses whether the same examiner can reproduce identical measurements across two sessions, was calculated using a two-way mixed-effects model with absolute agreement (ICC [1, 3]). This model is appropriate when the same examiners evaluate the same units. Inter-observer agreement, which assesses the consistency between different examiners measuring the same units, was calculated using a two-way random-effects model with absolute agreement (ICC [1, 2]) [15]. A paired two-sample t-test was used to compare normally distributed data according to the two methods, and a Wilcoxon test was used to compare non-normally distributed data. The significance level was set at *p <* 0.05.

## Results

ICCs were calculated to assess both intra- and inter-observer variability within each group, and the results were subsequently compared across groups.

ICC values were interpreted as follows: 0.0–0.50, low; 0.50–0.75, moderate; 0.75–0.90, good; and 0.90–1.00, high agreement [16, 17].

This classification was applied to evaluate intra- and inter-observer reliability. The overall intra-observer ICC exceeded 0.95, indicating excellent reliability (Table 2).

**Table 2:** Assessment of intra-observer and inter-observer agreement

	Researcher 1				Researcher 2				Researcher 1–2	
	Initial	Final	ICC (%95 CI)	*p*	Initial	Final	ICC (%95 CI)	*p*	ICC (%95 CI)	*p*
mx occlusal plane to TVL	97,63 ± 4,95	97,71 ± 5,11	0,968 (0,934–0,984)	< 0,001	97,61 ± 5,16	97,68 ± 5,32	0,988 (0,975–0,994)	< 0,001	0,959 (0,916–0,98)	< 0,001
mx 1 to mx occlusal plane	57,28 ± 5,32	56,70 ± 5,75	0,924 (0,85 − 0,962)	< 0,001	56,69 ± 6,09	56,25 ± 6,24	0,983 (0,966–0,992)	< 0,001	0,894 (0,795–0,947)	< 0,001
md 1 to md occlusal plane	66,95 ± 8,18	66,59 ± 7,52	0,946 (0,892–0,973)	< 0,001	66,73 ± 7,79	66,81 ± 7,70	0,987 (0,974–0,994)	< 0,001	0,955 (0,91 − 0,978)	< 0,001
overjet	3,57 ± 2,31	3,61 ± 2,15	0,977 (0,954–0,989)	< 0,001	3,70 ± 2,26	3,77 ± 2,24	0,992 (0,985–0,996)	< 0,001	0,951 (0,902–0,976)	< 0,001
overbite	2,70 ± 2,06	2,77 ± 2,00	0,955 (0,909–0,978)	< 0,001	3,22 ± 1,44	3,39 ± 1,51	0,967 (0,934–0,984)	< 0,001	0,447 (0,121–0,685)	0,005
upper lip thickness	13,05 ± 2,52	12,50 ± 2,79	0,961 (0,922–0,981)	< 0,001	12,53 ± 2,85	12,64 ± 2,77	0,988 (0,976–0,994)	< 0,001	0,943 (0,886–0,972)	< 0,001
lower lip thickness	14,20 ± 1,72	14,02 ± 1,68	0,665 (0,416–0,821)	< 0,001	13,98 ± 1,55	13,97 ± 1,66	0,923 (0,849–0,962)	< 0,001	0,72 (0,499–0,852)	< 0,001
pogonion-pogonion’	12,27 ± 2,13	11,99 ± 2,14	0,939 (0,879–0,97)	< 0,001	12,22 ± 1,86	12,19 ± 1,99	0,987 (0,974–0,994)	< 0,001	0,917 (0,837–0,958)	< 0,001
menton-menton’	8,38 ± 1,95	8,33 ± 2,32	0,841 (0,7 − 0,919)	< 0,001	8,47 ± 2,25	8,44 ± 2,23	0,992 (0,984–0,996)	< 0,001	0,85 (0,715–0,924)	< 0,001
nasolabial angle	107,20 ± 10,59	107,15 ± 10,66	0,988 (0,975–0,994)	< 0,001	107,28 ± 10,60	107,16 ± 11,05	0,991 (0,982–0,996)	< 0,001	0,958 (0,917–0,979)	< 0,001
upper lip angle	2,30 ± 9,60	2,47 ± 9,42	0,991 (0,982–0,996)	< 0,001	3,00 ± 9,57	3,56 ± 9,86	0,99 (0,98 − 0,995)	< 0,001	0,982 (0,964–0,991)	< 0,001
nasion’-menton’	123,27 ± 8,10	123,19 ± 8,63	0,947 (0,895–0,974)	< 0,001	121,78 ± 8,19	118,63 ± 20,66	0,325 (-0,022 − 0,601)	0,033	0,861 (0,734–0,93)	< 0,001
upper lip length	20,70 ± 2,90	20,48 ± 3,07	0,919 (0,84 − 0,959)	< 0,001	20,73 ± 3,13	20,55 ± 3,34	0,983 (0,966–0,992)	< 0,001	0,869 (0,749–0,934)	< 0,001
interlabial gap	0,85 ± 1,23	0,95 ± 1,36	0,873 (0,757–0,936)	< 0,001	0,92 ± 1,31	0,98 ± 1,35	0,987 (0,975–0,994)	< 0,001	0,863 (0,739–0,931)	< 0,001
lower lip length	46,64 ± 4,57	46,56 ± 4,90	0,928 (0,858–0,964)	< 0,001	46,97 ± 4,82	46,75 ± 5,12	0,982 (0,964–0,991)	< 0,001	0,89 (0,787–0,945)	< 0,001
lower 1/3 of face	67,66 ± 6,92	67,48 ± 7,42	0,951 (0,902–0,976)	< 0,001	69,19 ± 8,80	68,09 ± 7,22	0,762 (0,566–0,876)	< 0,001	0,773 (0,585–0,882)	< 0,001
Mx1 exposure	2,75 ± 2,04	2,80 ± 1,98	0,985 (0,969–0,993)	< 0,001	2,83 ± 2,03	2,86 ± 2,03	0,994 (0,988–0,997)	< 0,001	0,965 (0,93 − 0,983)	< 0,001
maxillary height	23,43 ± 3,69	23,42 ± 3,78	0,954 (0,907–0,977)	< 0,001	23,89 ± 3,89	23,94 ± 3,87	0,992 (0,984–0,996)	< 0,001	0,908 (0,82 − 0,954)	< 0,001
mandibular height	47,17 ± 4,01	46,86 ± 3,84	0,909 (0,822–0,955)	< 0,001	47,30 ± 3,64	47,30 ± 3,78	0,984 (0,967–0,992)	< 0,001	0,853 (0,721–0,926)	< 0,001
glabella	-6,18 ± 6,57	-5,88 ± 6,38	0,992 (0,984–0,996)	< 0,001	-5,23 ± 6,81	-5,25 ± 6,80	0,998 (0,996–0,999)	< 0,001	0,87 (0,751–0,934)	< 0,001
alar base	-12,50 ± 1,91	-12,44 ± 2,00	0,978 (0,953–0,989)	< 0,001	-12,25 ± 1,88	-12,19 ± 1,99	0,983 (0,966–0,992)	< 0,001	0,907 (0,813–0,954)	< 0,001
nasal projection	15,33 ± 1,90	15,50 ± 1,90	0,899 (0,803–0,949)	< 0,001	13,61 ± 8,41	13,55 ± 8,55	1 (0,999–1)	< 0,001	0,03 (-0,317–0,37)	0,434
subnasale	0,00 ± 0,00	0,00 ± 0,00	---	---	0,00 ± 0,00	0,00 ± 0,00	---	---	---	---
A point’	-2,05 ± 0,76	-2,05 ± 0,82	0,881 (0,77 − 0,94)	< 0,001	-1,95 ± 0,86	-1,97 ± 0,86	0,995 (0,989–0,997)	< 0,001	0,664 (0,415–0,821)	< 0,001
upper lip anterior’	0,67 ± 2,51	0,70 ± 2,48	0,984 (0,967–0,992)	< 0,001	0,41 ± 2,99	0,45 ± 2,99	0,997 (0,994–0,999)	< 0,001	0,784 (0,602–0,888)	< 0,001
Mx1	-12,58 ± 4,30	-11,45 ± 5,94	0,604 (0,329–0,785)	< 0,001	-11,67 ± 3,83	-10,86 ± 5,79	0,514 (0,206–0,729)	0,001	0,937 (0,876–0,969)	< 0,001
Mn1	-15,97 ± 4,88	-14,34 ± 7,52	0,4 (0,064 − 0,654)	0,011	-15,20 ± 4,40	-15,19 ± 4,46	0,995 (0,99 − 0,997)	< 0,001	0,959 (0,917–0,98)	< 0,001
lower lip anterior’	-1,82 ± 4,20	-1,62 ± 3,97	0,988 (0,976–0,994)	< 0,001	-1,61 ± 3,90	-1,55 ± 3,96	0,999 (0,998–1)	< 0,001	0,982 (0,964–0,991)	< 0,001
B point’	-9,95 ± 5,12	-9,84 ± 5,03	0,981 (0,961–0,99)	< 0,001	-10,06 ± 5,00	-10,06 ± 4,98	0,997 (0,995–0,999)	< 0,001	0,978 (0,954–0,989)	< 0,001
Pogonion’	-7,72 ± 5,96	-7,66 ± 5,84	0,989 (0,978–0,995)	< 0,001	-7,69 ± 5,56	-7,70 ± 5,68	0,998 (0,997–0,999)	< 0,001	0,979 (0,957–0,99)	< 0,001
facial angle	164,91 ± 6,97	165,02 ± 7,23	0,977 (0,953–0,989)	< 0,001	166,13 ± 7,02	166,50 ± 7,03	0,985 (0,97 − 0,993)	< 0,001	0,957 (0,915–0,979)	< 0,001
G’ to A’	5,20 ± 5,79	4,93 ± 5,69	0,979 (0,958–0,99)	< 0,001	4,78 ± 5,73	4,70 ± 5,81	0,999 (0,997–0,999)	< 0,001	0,984 (0,967–0,992)	< 0,001
G’ to Pog’	-0,15 ± 8,54	-0,57 ± 8,39	0,965 (0,93 − 0,983)	< 0,001	-0,80 ± 8,19	-1,06 ± 8,46	0,985 (0,97 − 0,993)	< 0,001	0,966 (0,932–0,983)	< 0,001
Sn to Pog’	7,92 ± 5,99	7,67 ± 5,59	0,975 (0,949–0,987)	< 0,001	7,56 ± 5,46	7,53 ± 5,45	0,998 (0,997–0,999)	< 0,001	0,98 (0,96 − 0,99)	< 0,001
A’ to B’	7,74 ± 4,72	7,92 ± 4,66	0,973 (0,946–0,987)	< 0,001	7,78 ± 4,54	7,83 ± 4,55	0,998 (0,997–0,999)	< 0,001	0,977 (0,953–0,989)	< 0,001
ULA to LLA	2,23 ± 2,77	2,65 ± 3,27	0,671 (0,426–0,825)	< 0,001	2,31 ± 2,66	2,36 ± 2,60	0,997 (0,994–0,999)	< 0,001	0,972 (0,944–0,986)	< 0,001
Md1 to Pog’	9,59 ± 8,69	8,19 ± 3,36	0,347 (0,003 − 0,617)	0,024	7,97 ± 3,44	7,98 ± 3,33	0,994 (0,988–0,997)	< 0,001	0,308 (-0,04 − 0,589)	0,041
LLA to Pog’	6,02 ± 3,65	5,98 ± 3,63	0,98 (0,959–0,99)	< 0,001	6,22 ± 3,40	6,23 ± 3,41	0,997 (0,994–0,998)	< 0,001	0,972 (0,944–0,986)	< 0,001
B’ to Pog’	-0,44 ± 2,75	-0,40 ± 2,80	0,987 (0,974–0,994)	< 0,001	-0,50 ± 2,72	-0,53 ± 2,77	0,998 (0,996–0,999)	< 0,001	0,908 (0,82 − 0,954)	< 0,001
NTP to Pog’	48,70 ± 6,12	48,83 ± 5,71	0,971 (0,942–0,985)	< 0,001	48,70 ± 5,89	48,76 ± 5,94	0,987 (0,973–0,993)	< 0,001	0,933 (0,868–0,966)	< 0,001

ICC (%95 CI): Intraclass correlation coefficient (%95 confidence interval), *Significance set at* *p < 0.05*

### Intra-observer agreement

For Researcher 1, ICC values for initial measurements range from 0.003 to 0.984, indicating low to excellent agreement, and for final measurements, from 0.617 to 0.996, indicating moderate to excellent agreement. The lowest intra-observer agreement was observed for mandibular incisor-soft tissue pogonion (Md1 to Pog’) (ICC = 0.347), while the highest agreement was found for Glabella (GB) (ICC = 0.984) (Table 2).

For Researcher 2, the ICC values ranged from 0.022 to 0.999 for initial measurements and 0.601 to 0.999 for final measurements. While the majority of measurements indicated good-to-excellent intra-observer reliability (ICC > 0.75), the observed minimum value of 0.022 suggests extremely poor agreement for that specific measurement point, necessitating a more cautious interpretation of the overall reliability. Exceptions were observed for the Nasion’–Menton’, lower one-third of the face, and Mx 1 exposure, which showed moderate to low agreement.

### Inter-observer agreement

Researcher 1 and Researcher 2 demonstrated statistically significant differences in 37 parameters, except for overbite, nasal projection, and Md1 to Pog *(p <* 0.05*)* (Table 2). Inter-observer ICC values ranged widely, from 0.03 to 0.984, but most measurements showed excellent agreement. Some exceptions were found for Mx occlusal plane to TVL, Mx 1 to Mx occlusal plane, Menton-Menton’, Nasion’-Menton’, upper lip length, interlabial gap, lower lip length, lower 1/3 face, upper lip anterior’ (ULA), which all showed very good inter-observer reliability (ICC ranging from 0.773 to 0.959). In contrast, moderate agreement was found for lower lip thickness and A point’ (ICC = 0.72, ICC = 0.664), and low agreement was observed for overbite and Mn1 to Pog’ (ICC = 0.447, ICC = 0.308). Nasal projection demonstrated poor reliability with no statistically significant agreement (ICC = 0.325, *p* = 0.434) (Table 2).

### Comparison of manual tracing and WebCeph Premium™

Manual tracings were compared with the WebCeph Premium™ AI system to assess measurement agreement. Measurements such as Mx 1 to Mx occlusal plane, Md 1 to Md occlusal plane, overjet, upper lip thickness, lower lip thickness, Pogonion-Pogonion’, Menton-Menton’, nasolabial angle (NLA), Nasion’-Menton’, lower 1/3 of face, Mx1 exposure, Mx height, Mandibular height, GB, upper lip anterior’, Mx1, Mn1, lower lip anterior’ (LLA), B Point’, Pogonion’, Facial Angle, G’ to A’, G’ to Pog’, Sn to Pog’, A’ to B’, ULA to LLA, Md1 to Pog’, LLA to Pog’, B’ to Pog’, NTP to Pog’ demonstrated excellent agreement between the two methods (ICC value between 0.913 and 0.995) (Table 3).

**Table 3:** Comparison of manual tracing and WebCeph™ using paired t-Test, Wilcoxon test, and intraclass correlation coefficient (ICC, 95% CI)

	WC		Manual		*p*	ICC (%95 CI)	*p*
	Mean ± S.D.	Median (min. - max.)	Mean ± S.D.	Median (min. - max.)			
mx occlusal plane to TVL	97,63 ± 5,08	96,52 (88,92–110,60)	97,62 ± 5,00	97,00 (88,00–110,75)	0,982*	0,989 (0,976–0,994)	< 0,001
mx 1 to mx occlusal plane	58,61 ± 5,71	58,02 (48,04–71,99)	56,98 ± 5,56	56,00 (47,00–69,50)	< 0,001*	0,952 (0,744–0,984)	< 0,001
md 1 to md occlusal plane	67,53 ± 8,17	67,20 (50,61–90,78)	66,84 ± 7,90	66,25 (52,50–89,50)	0,031*	0,987 (0,971–0,994)	< 0,001
overjet	3,65 ± 2,22	3,46 (0,00–12,10)	3,64 ± 2,26	3,38 (-0,50 − 12,50)	0,822**	0,992 (0,984–0,996)	< 0,001
overbite	2,84 ± 1,61	2,73 (0,27 − 6,75)	2,96 ± 1,51	3,00 (0,00–6,00)	0,538*	0,867 (0,728–0,935)	< 0,001
upper lip thickness	13,36 ± 2,44	13,46 (8,29 − 18,39)	12,79 ± 2,65	12,63 (8,00–17,25)	< 0,001*	0,965 (0,857–0,987)	< 0,001
lower lip thickness	14,27 ± 1,70	14,22 (10,78 − 17,29)	14,09 ± 1,52	14,00 (11,50 − 18,00)	0,196*	0,939 (0,876–0,97)	< 0,001
pogonion-pogonion’	12,76 ± 1,98	12,47 (9,28 − 16,98)	12,24 ± 1,96	12,13 (9,00–16,25)	< 0,001*	0,955 (0,799–0,984)	< 0,001
menton-menton’	8,51 ± 1,97	8,20 (4,94 − 12,34)	8,43 ± 2,03	8,00 (4,75 − 12,75)	0,368*	0,982 (0,963–0,991)	< 0,001
nasolabial angle	108,16 ± 11,17	107,75 (88,06–132,29)	107,24 ± 10,48	106,25 (87,00–130,50)	0,085*	0,98 (0,959–0,99)	< 0,001
upper lip angle	1,65 ± 9,22	1,44 (-13,35 − 17,90)	2,65 ± 9,55	2,00 (-11,75 − 23,00)	0,004*	0,988 (0,967–0,995)	< 0,001
nasion’-menton’	123,06 ± 8,93	122,40 (105,81–141,81)	122,52 ± 7,85	121,25 (105,00–141,50)	0,403*	0,953 (0,904–0,977)	< 0,001
upper lip length	21,27 ± 3,04	20,48 (16,32 − 29,04)	20,72 ± 2,92	20,13 (14,50 − 28,75)	0,005**	0,967 (0,903–0,986)	< 0,001
interlabial gap	0,83 ± 1,33	0,70 (-1,99 − 6,13)	0,89 ± 1,23	1,00 (-1,00–5,75)	0,525**	0,971 (0,942–0,986)	< 0,001
lower lip length	47,79 ± 4,59	47,50 (37,71 − 58,08)	46,80 ± 4,56	46,75 (36,50–57,25)	< 0,001*	0,975 (0,839–0,992)	< 0,001
lower 1/3 of face	69,88 ± 7,24	68,88 (53,75–87,66)	68,42 ± 7,45	67,00 (52,00–87,50)	0,026*	0,93 (0,846–0,967)	< 0,001
Mx1 exposure	2,87 ± 2,10	2,98 (-1,83 − 6,35)	2,79 ± 2,02	2,88 (-2,00–6,00)	0,275*	0,989 (0,978–0,995)	< 0,001
maxillary height	24,14 ± 3,74	24,17 (17,45 − 30,85)	23,66 ± 3,70	23,75 (15,50 − 31,00)	0,023*	0,972 (0,937–0,987)	< 0,001
mandibular height	47,74 ± 3,48	48,17 (41,83 − 57,02)	47,23 ± 3,69	47,50 (41,50–57,50)	0,101*	0,938 (0,872–0,97)	< 0,001
glabella	-6,66 ± 6,76	-7,51 (-17,45 − 12,27)	-5,71 ± 6,47	-6,00 (-16,50 − 11,25)	0,009*	0,974 (0,935–0,988)	< 0,001
alar base	-13,05 ± 2,19	-13,07 (-18,70 - -7,52)	-12,36 ± 1,86	-12,25 (-16,00 - -7,50)	0,005*	0,859 (0,661–0,936)	< 0,001
*nasal projection*	*16*,*05 ± 1*,*76*	*15*,*77 (13*,*02–20*,*02)*	*14*,*47 ± 4*,*38*	*15*,*13 (-1*,*00–20*,*00)*	*< 0*,001**	*0*,*325 (-0*,*284–0*,*658)*	*0*,*119*
subnasale	0,00 ± 0,00	0,00 (0,00–0,00)	0,00 ± 0,00	0,00 (0,00–0,00)	---	---	---
A point’	-2,18 ± 0,63	-2,23 (-3,39 - -0,84)	-2,00 ± 0,74	-2,00 (-3,50 - -0,75)	0,016*	0,891 (0,755–0,949)	< 0,001
upper lip anterior’	0,21 ± 2,98	0,43 (-8,00–5,88)	0,54 ± 2,60	0,75 (-4,25 − 6,00)	0,02*	0,979 (0,95 − 0,99)	< 0,001
Mx1	-13,45 ± 4,28	-14,11 (-20,90 - -4,20)	-12,13 ± 4,01	-12,38 (-19,50 - -4,00)	< 0,001*	0,958 (0,499–0,988)	< 0,001
*Mn1*	-16,73 ± 4,45	*-17,83 (-23,30 - -5,82)*	*-15,59 ± 4,60*	*-16,13 (-23,50 - -4,00)*	*<* 0,001**	*0,971 (0,716–0,991)*	< 0,001
lower lip anterior’	-2,13 ± 3,92	-1,97 (-9,66 − 7,28)	-1,71 ± 4,03	-1,69 (-9,50 − 8,00)	0,006*	0,987 (0,966–0,994)	< 0,001
B point’	-10,54 ± 5,25	-10,32 (-19,06 − 2,33)	-10,01 ± 5,03	-9,94 (-18,50 − 3,00)	0,006*	0,988 (0,968–0,995)	< 0,001
pogonion’	-8,23 ± 5,95	-8,01 (-18,43 − 4,07)	-7,70 ± 5,73	-7,69 (-18,00–5,00)	0,001*	0,993 (0,977–0,997)	< 0,001
facial angle	164,76 ± 7,15	164,88 (150,91–177,80)	165,52 ± 6,92	165,00 (149,50–177,50)	0,004*	0,988 (0,966–0,995)	< 0,001
G’ to A’	5,24 ± 5,96	5,47 (-7,39 − 15,56)	4,99 ± 5,73	5,75 (-8,00–15,00)	0,085*	0,995 (0,989–0,998)	< 0,001
G’ to Pog’	-0,39 ± 8,67	-0,70 (-21,88 − 18,27)	-0,47 ± 8,30	-0,50 (-21,50 − 17,50)	0,719*	0,995 (0,989–0,997)	< 0,001
Sn to Pog’	8,41 ± 5,97	8,49 (-4,07–18,43)	7,74 ± 5,70	7,88 (-5,00–18,00)	< 0,001*	0,991 (0,959–0,997)	< 0,001
A’ to B’	8,27 ± 4,87	8,32 (-3,78 − 16,48)	7,76 ± 4,60	8,00 (-3,75 − 16,50)	< 0,001*	0,991 (0,966–0,997)	< 0,001
ULA to LLA	2,36 ± 2,72	2,82 (-3,33 − 8,53)	2,27 ± 2,70	2,56 (-3,25 − 8,50)	0,320*	0,992 (0,984–0,996)	< 0,001
Md1 to Pog’	9,82 ± 8,97	8,82 (0,00–54,13)	8,78 ± 5,34	8,25 (1,00–31,00)	0,038**	0,913 (0,822–0,957)	< 0,001
LLA to Pog’	6,10 ± 3,62	5,87 (-1,91 − 12,52)	6,12 ± 3,50	5,75 (-1,50 − 14,50)	0,883*	0,99 (0,98 − 0,995)	< 0,001
B’ to Pog’	-0,36 ± 2,86	-0,81 (-5,45 − 5,65)	-0,47 ± 2,67	-0,75 (-5,50 − 5,63)	0,390*	0,984 (0,967–0,992)	< 0,001
NTP to Pog’	48,53 ± 6,05	49,56 (35,25–61,90)	48,70 ± 5,91	49,50 (35,00–59,50)	0,517*	0,986 (0,972–0,993)	< 0,001

*Paired t test, **Wilcoxon test, ICC (%95 CI): Intraclass Correlation Coefficient (%95 95% CI). *Significance set at* *p < 0.05*

This high level of agreement underscores the reliability of the WebCeph Premium™ system in performing cephalometric analyses that are comparable to those of manual techniques. Minor variations were observed in certain parameters, including overbite, alar base, and A point, which demonstrated good agreement (ICC = 0.867, 0.859, 0.891, respectively). In contrast, nasal projection showed poor reliability (ICC = 0.325) (Table 3).

## Discussion

Numerous studies have compared the accuracy and reproducibility of cephalometric analyses performed through manual tracing and computer-assisted software. Recent research has increasingly focused on evaluating the accuracy and reproducibility of AI-driven cephalometric applications [6, 18–24]. The aim of our study was to evaluate whether AI-supported cephalometric programs for Arnett analysis—a widely used cephalometric method globally as a standard in the diagnosis and treatment planning of orthognathic surgery—can positively contribute to clinical practice for orthodontists.

This study evaluates the accuracy of linear and angular cephalometric values generated by the WebCeph Premium™ application for Arnett analysis by comparing them with those obtained through manual tracing. We focused on cephalometric measurements rather than landmark identification, as these directly influence treatment decisions and reduce potential errors from landmark variability [25]. Inaccuracies in AI-based cephalometric analysis may result from improper head positioning, radiographic distortions, and calibration errors (manual in Ceppro DDH Inc. and automatic in WebCeph™). In addition, the reliability of landmark identification is influenced by image quality, orthodontic training, and the clinician’s level of experience in performing cephalometric measurements with AI-supported tools [20]. Accuracy varies by anatomical complexity, with lower intra-observer errors than inter-observer discrepancies [8, 26]. Analysis confirmed that repeatability varies based on anatomical structures, with clear landmarks (e.g. Glabella, 0.984) demonstrating excellent repeatability. However, measurements involving indistinct landmarks (e.g. Md1-Pog’, 0.347) showed lower agreement. Even experienced observers struggle to determine soft tissue, highlighting the challenges of these measurements. Moderate-to-low agreement in specific regions, like the lower one-third of the face, shows that measurements critical to aesthetic assessments are highly susceptible to intra-observer error. These results show that a single manual measurement for these low repeatability parameters in aesthetic treatment planning should be avoided. Automated landmark detection in digital systems reduces this variability and enhances reproducibility. In the present study, AI-determined landmarks were retained for objective evaluation of the algorithm’s performance.

AI-driven tools automatically identify cephalometric landmarks, significantly accelerating analysis.^4^ Studies show AI-assisted software reduces tracing time and improves accuracy, with meta-analyses confirming its effectiveness in 2D and 3D-CBCT imaging [27]. Katyal et al. reported that WebCeph™ was faster and more reliable than semi-automated FACAD and manual tracing, suggesting that it represents a practical tool for cephalometric analysis [28].

Numerous statistical methods, including the Pearson correlation coefficient, the paired t-test, the Bland-Altman plot, and the ICC, routinely used to assess the reliability of diverse measurements. Zaki et al. reviewed statistical techniques for evaluating medical instruments and identified ICC as the most widely used method for continuous data [29]. Kotula et al. applied ICC to evaluate the repeatability and reproducibility of newly defined cephalometric landmarks for the ANB, Tau, and Yen angles [15]. They used a two-way mixed-effects model for intra-observer agreement (ICC (3,1)) and a two-way random-effects model for inter-observer agreement (ICC (2,1)). Accordingly, ICC was applied to assess the agreement between manual tracing and WebCeph Premium™, thereby enhancing the methodological rigor of the study and providing an objective demonstration of the reliability of AI-based measurements.

Consistency between and within examiners is critical in cephalometric analysis, as landmark inaccuracies can lead to errors [30, 31]. Inter-examiner discrepancies are generally greater than intra-examiner variations [32]. In line with this, Kim et al. highlighted that examiner-related variability is crucial for evaluating the effectiveness of AI landmark detection [22]. In this double-blind crossover study, two orthodontists evaluated 5 images per session to minimize fatigue-related bias. Individuals exhibiting craniofacial asymmetry were excluded from our study. Asymmetric cases can lead to errors in cephalometric measurements and heterogeneity within the sample by affecting the positions of bilateral anatomical reference points. Therefore, only symmetrical individuals were included in the study to enhance the accuracy of the measurements and the reliability of the intra-group comparisons [33].

The study revealed that, despite non-significant p-values, certain parameters demonstrated high ICC values. These parameters included the maxillary occlusal plane to TVL, overjet, overbite, lower lip thickness, Menton-Menton’, NLA, Nasion’-Menton’, interlabial gap, maxillary incisor exposure, mandibular height, G’ to Pog’, upper lip to lower lip angle, lower lip to Pog’, B’ to Pog’, and NTP to Pog’. These findings reflect strong agreement; however, they also highlight possible limitations, including a relatively small sample size and inherent data variability. The confidence interval (CI) is more informative than the p-value, as it reflects the true population ICC range. Conversely, nasal projection exhibited a low ICC value but a significant p-value (*p* < 0.001), highlighting that statistical significance alone does not guarantee reliability. Therefore, the ICC should always be interpreted in conjunction with its CI. Although gender-specific analyses were not performed, the equal representation of men and women ensures that the results reflect a balanced sample, thereby reducing potential sampling bias.

Manual cephalometric analysis is subjective, as variations in orthodontists’ landmark localization can result in discrepancies of up to 1.5 mm. Even repeated measurements by the same clinician can result in errors of up to 1 mm. In contrast, AI algorithms offer consistent and precise landmark identification [23]. In the current study, the initial ICC values for Researcher 1 ranged from 0.003 to 0.984, with the final measurement improved to 0.996, thereby demonstrating moderate to excellent consistency. Similarly, for Researcher 2, the initial ICC values ranged from 0.022 to 0.999, and the final measurement reached 0.999, indicating low to excellent intra-observer reliability.

Çoban et al. evaluated 23 cephalometric landmarks on 105 X-rays using both manual tracing and WebCeph™ software [6]. Their analysis revealed statistically significant differences between the two methods. Notable discrepancies were observed in the measurements of SNA, ANB, NA, Y-axis, SN, Go-Gn, SN-PP, ANS-Me, Co-A, Co-Gn, U1-PP, U1-NA, IMPA, L1-NB, L1-NB, NLA and upper lip to esthetic line (ULE).

Mahto et al. assessed 18 cephalometric landmarks and 12 angular and linear measurements on 30 X-rays using both manual tracing and WebCeph™ [4]. They reported high ICC values for 7 out of the 12 measurements, including ANB, FMA, IMPA, LL- E line, L1- NB (mm), L1- NB (degrees), and S-N to Go-Gn. For the subsequent five measurements, the ICC values ranged between 0.75 and 0.9. Tsolakis et al. compared automatic landmark identification using CS Imaging V8 with manual tracing on 100 cephalometric X-rays. While mean values demonstrated no statistically significant differences, substantial discrepancies were observed in specific measurements: FMA (2.1°), IMPA (4°), ANS-PNS/Go-Gn (3°), and U1-L1 (3.3°) [34].

Our evaluation comparing manual tracing with WebCeph Premium™ revealed excellent agreement (ICC: 0.913–0.995) for most parameters, including dental relationships (Mx1/Md1 to occlusal planes), facial measurements (NLA, facial height), and soft tissue analyses (lip thickness, pogonion’). Although challenging landmarks such as gonion and porion have shown variability in previous studies, WebCeph™ demonstrated consistent performance across most measurements [2, 35, 36]. Our study’s results show the high consistency of most soft tissue landmarks, making them useful in orthodontic treatment and orthognathic surgery planning. Clinicians can rely on the stability of these measurements for aesthetic decisions. There is slightly lower but still strong agreement (ICC: 0.867–0.891) for overbite, alar base and A point, suggesting these parameters may benefit from additional refinement. The system’s high reproducibility supports its clinical utility as an alternative to manual tracing.

As demonstrated in previous studies, AI cephalometric analysis is associated with a high level of reliability. In particular, Silva et al. reported ICCs of 0.768–0.997 for CEFBOT, and Panesar et al.^36^ found 0.890-1.00 for 26 measurements [13]. In comparison, the present study yielded ICCs of 0.867–0.995 across 40 measurements using WebCeph Premium™, with statistically significant differences in 37 parameters, indicating high accuracy relative to manual tracing.

The identification of cephalometric landmarks, such as the gonion, porion, menton, gnathion, orbitale, articulare, lower incisor apex and A point, is often challenging [2, 35, 36]. Panesar et al. found four measurements, including A point, showed moderate accuracy without AI, mainly due to repeatability challenges [37]. Point A is difficult to locate due to its proximity to the incisor root, overlapping structures, indistinct anterior maxillary curvature, and the nearby fossa canine. While A point exhibited good reliability on the x-axis, significant variability was observed on the y-axis. In our study, A point demonstrated good agreement (ICC = 0.891) [38].

Arik et al. explored the potential of AI to reduce measurement errors by comparing manual and AI-driven measurements [7]. They found that AI minimized manual digitization errors and provided consistent results. Kim et al. used a deep learning system to identify 20 landmarks in 3150 lateral cephalograms, achieving 90% accuracy (mean error of 1.5 mm) [39]. This suggests that AI can reduce variability in identifying landmarks, especially in routine cases. Ye et al. assessed 43 lateral cephalograms using AI-based programs (MyOrthoX, Angelalign, and Digident), finding significant time savings compared to manual methods due to differences in landmark identification techniques [40]. All programs achieved accuracy rates above 85% with a clinically acceptable precision threshold of 2 mm.

Nasal projection showed low reliability (ICC = 0.325) compared with previous studies [41]. This discrepancy likely reflects differences in landmarks, measurement techniques, or imaging modalities. The low ICC indicates variability in reference point definition, whereas the higher values in prior research highlight the precision of advanced 3D imaging. The low correlation of the overbite, nasal projection, and Md1 to Pog parameters, which are important in soft tissue assessment and determining the need for vertical control in occlusal adjustment, suggests that these landmarks should be clinically confirmed by orthodontists. Digital tools offer convenience but vary in reliability, so their use in practice should be approached with caution.

Kunz et al. found fewer landmark placement errors with AI than manual analysis of 150 lateral cephalograms, especially for challenging landmarks such as porion and condylion [42]. This demonstrates AI’s reliability in addressing anatomical variability and operator-dependent errors. The present study demonstrated moderate to high intra- and inter-observer agreement. Errors within 1 mm and deviations of less than 2° or 2 mm are generally considered to be clinically acceptable; however, Schwendicke et al. noted that cumulative errors in critical parameters, such as the ANB angle, can be significant. Manual tracing enables operators to exercise judgement and mitigate such inaccuracies [43].

### Limitations

This study had several limitations.

Restricting the sample to participants aged 16 and older improves measurement reliability; however, this criterion limits the generalizability of the study’s findings.

Moderate agreement for parameters such as overbite, alar base, and A point indicates that AI systems may encounter challenges in accurately assessing subtle anatomical features.

In Arnett’s analysis, metallic markers are placed to visualize soft tissue landmarks; however, this study did not employ the technique, thus excluding landmarks such as the orbital rim, cheekbone, subpupil, neck-throat junction, and alar base from manual measurements.

AI performance depends on the quality of training data and requires a robust technical infrastructure. Clinicians must critically interpret results, even in the face of automation.

### Further research directions

Future studies could expand the sample to include different age groups, sex distributions, and types of malocclusions to enhance the generalizability of the findings. Collecting data from multiple centers (multicentric studies) may further strengthen the external validity of the results. Additionally, besides WebCeph Premium™, other AI-based cephalometric software such as OneCeph, AudaxCeph^®^, MyOrthoX, and Digident could be tested for Arnett analysis. For measurements demonstrating lower reliability, detailed investigations could explore the anatomical challenges faced by AI algorithms, providing guidance for software improvements. Moreover, the development of AI systems could benefit from continuous learning through machine learning feedback, allowing algorithms to improve their performance over time.

## Conclusions

1. WebCeph Premium™ showed moderate to high reliability in cephalometric analysis.
2. Comparisons with manual tracing revealed that WebCeph Premium™ achieved high agreement for the majority of parameters, with minor discrepancies, thus confirming its reliability relative to traditional methods.
3. WebCeph Premium™ demonstrates comparable reliability to manual measurements for most cephalometric evaluations, suggesting its potential to enhance diagnostic efficiency in both clinical and research settings.
4. Future research should focus on parameters with lower levels of agreement and explore the applicability of WebCeph Premium™ in a range of clinical contexts to further validate its role in modern orthodontics.
5. AI development should continue to evolve, using the precision of the human eye as a benchmark for improvement.

## Supplementary Information

Below is the link to the electronic supplementary material.

Supplementary Material 1

Supplementary Material 2

## Data Availability

The datasets analyzed during the current study are not publicly available due to ethical restrictions but are available from the corresponding author on reasonable request.
