# Supplementary figures and images for: Comparison of W. Arnett’s cephalometric analysis measurements performed using artificial intelligence and manual measurements
Source: Clin Oral Investig. 2026 May 11;30(6):223. doi: 10.1007/s00784-026-06915-7 (PMC13160989; doi:10.1007/s00784-026-06915-7)

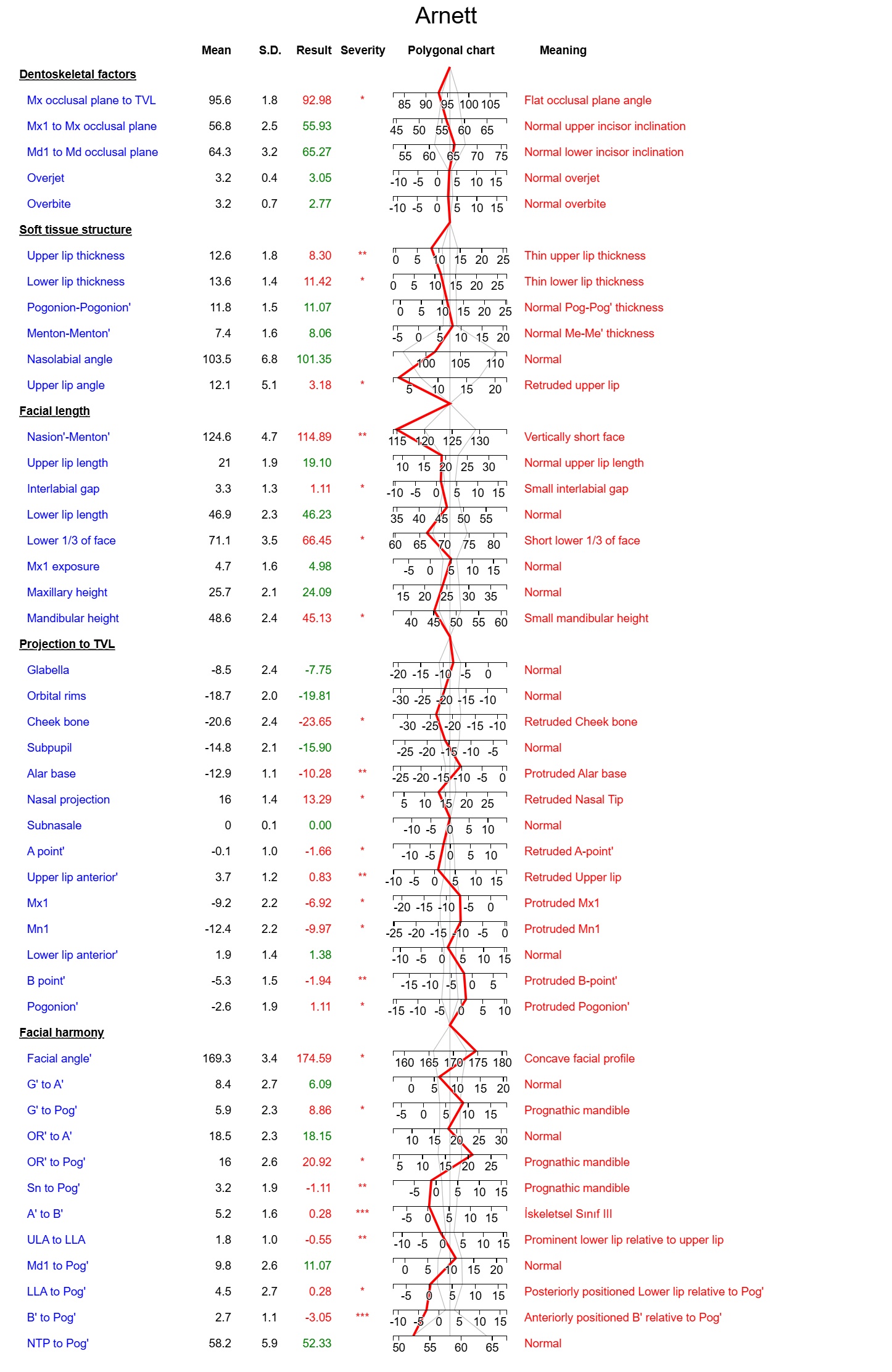

Supplement: Supplementary file 2 — Supplementary Material 2 [file 784_2026_6915_MOESM2_ESM.jpg]
